# Immune Thrombocytopenia and Type 1 von Willebrand Disease in a Patient With a Femoral Fracture: A Case Report

**DOI:** 10.7759/cureus.98215

**Published:** 2025-12-01

**Authors:** André Ferreira, André Roberto

**Affiliations:** 1 Anesthesiology, Unidade Local de Saúde de São José - Centro Hospitalar Universitário de Lisboa Central, Lisbon, PRT

**Keywords:** case report, femoral fracture, hemorrhagic risk, thrombocytopenia, von willebrand disease

## Abstract

This article describes the case of a complex medical patient with immune thrombocytopenia (ITP) and von Willebrand disease (vWD), among other comorbidities, who underwent urgent orthopedic surgery and discusses the specificities of the perioperative anesthetic management of these bleeding disorders. Immune thrombocytopenia is an acquired autoimmune condition characterized by a diminished platelet count due to immune destruction. vWD is the most common inherited bleeding disorder, in which there is a defect of von Willebrand factor, a fundamental component of the hemostatic process. By compromising primary hemostasis, both conditions carry an important hemorrhagic risk in surgical patients, which is compounded by their coexistence.

## Introduction

We present a case that depicts the successful perioperative management of a patient with type 1 von Willebrand disease (vWD) and immune thrombocytopenia (ITP) who underwent a non-cemented right hip hemiarthroplasty for a subcapital right femur fracture. Hip surgery carries a well-established bleeding risk and an increased need for allogeneic red blood cell (RBC) transfusions in the perioperative period [1-3], which can be further exacerbated by comorbid conditions and medications.

vWD is the most common inherited bleeding disorder, affecting about 1 in 100 people; nevertheless, it is asymptomatic in the majority of patients, being clinically significant in only 1 in 10,000 patients [4]. This hemostatic defect is the result of a defective von Willebrand factor (vWF), a plasmatic glycoprotein essential for normal platelet adhesion and aggregation, as well as for the maintenance of factor VIII levels. This defect can be either quantitative (low levels in type 1 and complete absence in type 3) or qualitative (impairment of action in type 2) [4-6]. As a consequence of an abnormal primary hemostasis, vWD predisposes patients to cutaneous and mucosal bleeding, easy bruising, frequent and intense epistaxis, heavy menstrual bleeding, anemia, and joint pain [5].

ITP is an acquired autoimmune type of thrombocytopenia, resulting from peripheral destruction and impaired megakaryopoiesis [7-10]. This condition configures a variable bleeding risk depending not only on the absolute platelet count but also on multiple other patient factors [7,11,12].

Both conditions compromise primary hemostasis, thus carrying significant hemorrhagic risk for surgery, requiring careful multidisciplinary management and optimization of the patient [7,11,13-15].

## Case presentation

A 77-year-old woman was admitted to the hospital after being diagnosed with a non-recent subcapital right femur fracture, with the intent of performing a hemiarthroplasty of the right hip. The patient was categorized as American Society of Anesthesiologists (ASA) IV [16], had a known allergy to penicillin, and her past medical history included splenic non-Hodgkin lymphoma (currently in remission after six cycles of R-CHOP chemotherapy); vWD (type 1, vWF:ristocetin cofactor (RCo) of 30.1% at diagnosis) and ITP (baseline platelet count of 40,000/μL); ischemic stroke (two years before the current episode, resulting in left central facial palsy and hemiparesis) and a previous transient ischemic stroke; colorectal cancer (subjected to a curative sigmoidectomy 10 years before); hypertension; first-degree atrioventricular block; dyslipidemia; hypothyroidism; peripheral venous disease; and depression. Surgical history included several dental extractions under local anesthesia, complicated with significant gingival bleeding, and a sigmoidectomy under general anesthesia, with no history of anesthetic complications or excessive bleeding, having received prophylactic vWF concentrate (Haemate P). Additionally, the patient had a history of several episodes of easy bruising and spontaneous gingival and rectal bleeding (years before the ITP diagnosis). Furthermore, she had a history of positive response to desmopressin and received vWF concentrate on several occasions. Current medication included lisinopril, amlodipine, furosemide, pravastatin, fenofibrate, lansoprazole, levothyroxine, diazepam, clonazepam, and fluoxetine.

On admission, she presented with pancytopenia, notably having a platelet count of 12,000/μL, thus being considered unfit for surgery. One platelet pool was transfused (yielding a value of 22,000/μL three days later), and the case was discussed with Hematology and Transfusion Medicine. A presumptive diagnosis of ITP was made, given her prior history of ITP with favorable response to corticosteroids and the absence of any indication of lymphoma progression. The patient was started on prednisolone 60 mg per day, and surgery was postponed until a platelet count above 50,000/μL was attained. Based on hemostatic levels of vWF and factor VIII (vWF:RCo = 294.3 IU/dL; FVIII:C = 248.1 IU/dL), it was decided there was no need to transfuse such factors. The administration of tranexamic acid 1 g and the transfusion of one platelet pool immediately before the surgery were also recommended.

After seven days of corticotherapy, the patient’s platelet count improved to 64,000/μL, and the levels of vWF and factor VIII remained at safe levels (vWF:RCo = 264.0 IU/dL; FVIII:C = 271.6 IU/dL); therefore, the patient was cleared for surgery.

As suggested, the following day, one platelet pool and 1 g of tranexamic acid were administered, and the patient underwent a non-cemented hip hemiarthroplasty under balanced general anesthesia. Standard ASA monitoring plus bispectral index monitor and train-of-four Neurostimulation testing was used, and two peripheral venous accesses (18 G) were established. Pre-oxygenation with 100% oxygen at 8 L/minute through a facial mask was performed for three minutes, followed by induction with fentanyl 0.1 mg, propofol 80 mg, and rocuronium 70 mg. The airway was then secured with a 7.0 endotracheal tube through direct laryngoscopy, and the patient was placed on volume-controlled ventilation. Hypnosis was maintained with sevoflurane, analgesia with fentanyl boluses (0.15 mg total), and neuromuscular blockade with rocuronium boluses (130 mg total). The patient was then placed in the left lateral decubitus position. Given that the patient had been receiving high-dose corticosteroids, an intraoperative stress dose of hydrocortisone 25 mg was administered before incision, followed by a 100 mg perfusion in the following 24 hours. Clindamycin 900 mg was administered as antibiotic prophylaxis. An arterial line was placed to allow for serial arterial blood gas analysis and better monitoring of blood loss.

In the intraoperative period, respiratory and hemodynamic stability were maintained, coagulation times and fibrinogen stayed normal (prothrombin time 11.6 seconds; activated partial thromboplastin time, 22.2 seconds; fibrinogen, 2.4 g/L), and platelet count remained stable (72,000/μL). Blood loss was estimated at 300 mL, and hemoglobin dropped from 7.9 to 7.2 g/dL intraoperatively; hence, one unit of RBC was transfused, yielding a final hemoglobin level of 8.8 g/dL. A total of 750 mL of polyelectrolytic IV fluid was infused; urine output was 600 mL. Ondansetron 4 mg was given for postoperative nausea and vomiting prophylaxis. As analgesia, paracetamol 1 g, tramadol 100 mg, and metamizole 2 g were administered. Nonsteroidal anti-inflammatory drugs (NSAIDs) were avoided because of the already high hemorrhagic risk. In addition, an ultrasound-guided femoral nerve block, with 20 mL of 0.375% ropivacaine, was also performed at the end of the procedure. After the surgery, the patient had an uneventful emergence from anesthesia.

Postoperatively, the patient was stable and transferred to a Level 2 intensive care unit bed for monitoring in the first 24 hours of the postoperative period. According to Transfusion Medicine’s suggestion, tranexamic acid 500 mg IV was administered every eight hours for the first 24 hours postoperatively. In the days that followed, she remained hemodynamically stable without any apparent bleeding. On day one postoperatively, one unit of RBC was transfused after a value of 6.5 g/dL of hemoglobin was obtained, and the patient was transferred to the infirmary. Despite needing two more units of RBCs (day two postoperatively), she remained clinically stable during her remaining time at the hospital. Venous thromboembolism prophylaxis with low-molecular-weight heparin was started on day three postoperatively once hemoglobin values stabilized. On day eight postoperatively, she presented a hemoglobin value of 8.7 g/dL and a platelet count of 44,000/μL. As she remained clinically stable, she was discharged from hospital care.

Table 1 presents a summary of laboratory findings recorded throughout the patient’s hospital course.

**Table 1:** Relevant laboratory results throughout the perioperative period. RBC = red blood cell; vWF = von Willebrand factor; RCo = ristocetin cofactor; FVIII = factor VIII

Parameters/Timing	At admission	Day 3 after admission	Day 10 after admission	Intraoperative period	Day 1 after surgery	Day 2 after surgery	Day 8 after surgery (discharge)	Reference range
Hemoglobin (g/dL)	10.3	9.2	7.9	7.2 (→ 8.8 after 1 RBC unit)	6.5 (→ 8.2 after 1 RBC unit)	6.7 (→ 9.7 after 2 RBC units)	8.7	12.0–15.0
Leucocytes (×10^9^/L)	3.32	2.55	2.87	2.98	3.73	2.79	2.28	4.5–11.0
Platelets (/µL)	12,000	22,000	64,000	78,000	74,000	71,000	44,000	150,000–450,000
Prothrombin time (seconds)	12.4	12.0	11.8	10.9/11.6	11.8	11.9	-	9.4–12.5
Activated partial thromboplastin time (seconds)	28.4	28.8	25.5	23.0/22.2	22.4	22.4	-	25.1–36.5
Fibrinogen (g/L)	-	-	-	2.4	2.2	-	-	2.00–4.00
vWF:Rco (%)	-	294.3	264.0	-	-	-	-	60.8–239.8
vWF:Ag (%)	-	233.5	235.7	-	-	-	-	66.1–176.3
FVIII:C (%)	-	248.1	271.6	-	-	-	-	50–150
Glucose (mg/dL)	105	82	79	81	73	85	102	60–100
Serum creatinine (mg/dL)	1.43	1.73	1.49	-	1.38	1.32	1.11	0.57–1.11
Serum urea (mg/dL)	77	63	102	-	84	68	74	21.0–43.0
Serum sodium (mEq/L)	140	135	136	138	139	141	139	136–145
Serum potassium (mEq/L)	3.7	4.0	4.5	4.2	4.4	4.3	4.1	3.50–5.10
Serum chloride (mEq/L)	104	96	100	104	107	109	107	98.0–107

## Discussion

Hip fractures, surgical treatment, and hemorrhagic risk

A hip fracture, or proximal femoral fracture, constitutes a fracture of the femur immediately distal to the hip joint and proximal to about 5 cm below the lower border of the lesser trochanter [17]. Most of these fractures are surgically treated through fixation with screws, pins, and plates or replacement of the femoral head with a prosthesis [17].

The increased bleeding risk associated with orthopedic surgery has been extensively documented by numerous studies, especially spine surgery and arthroplasty surgery [1-3]. In routine total hip arthroplasty (THA), studies have reported a wide range of prevalence of allogeneic RBC transfusions, namely, between 21% and 70% of THA patients [1-3]. As expected, blood loss in hip surgery is higher in vWD patients, both intraoperatively and postoperatively [18]. A retrospective case-controlled study found that vWD patients undergoing primary hip surgery had a higher calculated blood loss and drop in hemoglobin concentration when compared to their control counterparts, along with requiring more blood transfusions [19]. Thus, these patients require special attention to minimize blood loss and transfusion requirements.

von Willebrand factor: physiology

vWF, a plasmatic glycoprotein involved in primary hemostasis [4,5], is produced in megakaryocytes and endothelial cells in the form of multimers of variable size [4]. The majority are small and secreted into the plasma, being activated by collagen binding after tissue damage and becoming able to bind platelets, contributing to their adhesion and activation [4,5]. The larger multimers, the most active form, are stored in cytoplasmic granules and released as a physiological response to tissue damage mediators. Desmopressin, a synthetic analog of vasopressin, also triggers their release [4]. vWF also plays an important role in secondary hemostasis, acting as a factor VIII carrier, preventing its inactivation and allowing for the maintenance of hemostatic plasmatic levels [4].

von Willebrand disease: pathophysiology, presentation, and classification

As previously stated, vWD is a common inherited bleeding disorder in which vWF function is altered, quantitatively or qualitatively. This deficiency presents with various levels of easy bruising, mucosa-associated bleeding (particularly epistaxis, gingival, and gastrointestinal hemorrhage), and bleeding after surgery and trauma [5]. The vast majority of cases are autosomally inherited, but a rare acquired form of vWD has been described [5].

Based on phenotypic, laboratory, and genetic profiles, vWD is classified by the International Society on Thrombosis and Hemostasis into three main types. Type 1, the most frequent type (70% to 80% of all cases), is characterized by a quantitative deficiency (20% to 50% of normal values), with a wide spectrum of clinical presentations, varying from asymptomatic individuals to severe hemorrhage episodes [5]. Type 2 (15% to 30% of patients) represents a group of qualitative deficiencies, being further divided into four types based on the characteristics of vWF multimers [5]. Type 2A and 2M are characterized by decreased platelet-dependent vWF activity, the first having a deficit of high-molecular-weight multimers [5,13]. In type 2B, an increased binding to GPIba is found, leading to increased platelet clearance and, ultimately, thrombocytopenia [13]. Type 2N results from loss of factor VIII binding capability, leading to severe factor VIII deficiency and resembling a hemophilia A phenotype [5,13]. In type 3, there is a complete absence of detectable vWF, thus being the most severe form of vWD, which combines traits from vWD (decreased platelet function) and hemophilia A (factor VIII deficiency) [5,13].

Several factors can increase vWF and factor VIII plasma levels, such as systemic inflammation, pregnancy, increased estrogen levels, stress [13], and, most interestingly, age [14]. In fact, vWF and factor VIII plasma levels have been shown to increase with age; consequently, some of these patients’ levels may cross into a normal range later in life [14]. Nonetheless, recent studies seem to indicate that this increase may not necessarily be associated with a bleeding risk reduction [14]; hence, care must be taken even with normal factor levels.

von Willebrand disease: perioperative therapeutic agents

Perioperative therapeutic interventions in vWD can essentially be categorized into the following four strategy groups: (1) increase vWF and factor VIII levels by stimulating their release from endogenous storage (desmopressin); (2) improve hemostasis by inhibiting fibrinolysis (tranexamic or aminocaproic acids); (3) replacement of factor levels with exogenous vWF and/or factor VIII concentrates (alternatively, frozen fresh plasma or cryoprecipitate, if specific concentrates are not available); and (4) rescue measures (platelet infusion and recombinant factor VIIa) [13,14].

Desmopressin, a synthetic vasopressin analog, stimulates the release of vWF from platelets and endothelial cells through its agonist effect on V2 receptors [5,13,14]. After administration, baseline vWF and factor VIII levels are expected to increase three to fivefold [13,14]. This responsiveness is, however, dependent on the type of vWD. Generally, most patients with type 1 vWD respond to desmopressin, whereas patients with type 3 vWD do not. Type 2A, 2N, and 2M patients tend not to respond. In type 2B, desmopressin is relatively contraindicated, as it can transiently aggravate the thrombocytopenia [13,14]. In candidates for desmopressin administration, it is reasonable to test said response preoperatively [20]. If testing is unfeasible, type 1 adult patients with baseline vWF >0.30 IU/mL can be presumed responsive [6,13]. Desmopressin can be administered intravenously, subcutaneously, or intranasally. Its standard intravenous dose is 0.3 µg/kg (maximum 20 µg), diluted in 50 mL of normal saline and infused over 20 to 30 minutes, with a peak effect at 60 minutes and a response duration of 6-12 hours (depending on individual vWF clearance rates) [13,18]. Therefore, it should be given 90 minutes before the surgical procedure and can be repeated 8-12 hours later. Subsequent doses are usually less effective due to rapid storage depletion [13].

Antifibrinolytics, such as tranexamic acid and epsilon aminocaproic acid, are synthetic derivatives of the amino acid lysine that bind competitively to plasminogen, preventing its binding to fibrin and the subsequent conversion to plasmin, ultimately inhibiting fibrin degradation (fibrinolysis), thereby stabilizing blood clots. Tranexamic acid is the most potent and the most widely used; it can be administered topically, orally, or intravenously [13,14,21]. The intravenous dose of tranexamic acid is typically 10 to 15 mg/kg every eight hours, although some disparity in dosing regimens exists. Normally, a loading bolus of 1 g is administered over 10-15 minutes before surgery. A maintenance infusion can be administered over the following eight hours [13,14,21]. Its plasma half-life is about two hours, and it is mostly renally cleared [14]. Tranexamic acid is contraindicated in individuals with hypersensitivity to tranexamic acid, active arterial or venous thrombosis, a history of seizures, severe renal dysfunction, and in upper urinary tract bleeding [14,21]. Tranexamic acid has been shown to be effective in reducing blood loss and mortality in a myriad of scenarios, including trauma and orthopedic surgery [14,21-24]. Furthermore, its efficacy has also been demonstrated for vWD patients either on its own or as an adjuvant [14,25]. Therefore, it should be considered in all patients with vWD undergoing surgery (in the absence of contraindications) [14].

Factor replacement therapy is the gold standard treatment in vWD patients who do not respond to desmopressin or in whom it is contraindicated [13,14]. There are multiple vWF preparations, most derived from virally inactivated pooled plasma (pdvWF), containing not only vWF but also factor VIII. Two notable exceptions are worth mentioning: there is a pdvWF concentrate with almost no factor VIII (Wilfactin®), and recently, a recombinant vWF concentrate (rvWF) has been developed, also having no factor VIII (Vonvendi® /Veyvondi®) [13,14]. It should be noted that higher factor VIII content can elevate the thrombotic risk [13,14]. Thus, in patients with normal or near-normal factor VIII levels, the use of factor VIII-less concentrates should be considered [13].

The concentrate’s loading dose should be individualized to the patients’ levels and body weight, following the product’s recommended dosage. Generally, 1 IU/kg of vWF is considered to increase plasma vWF:RCo by approximately 2 IU/dL [13,14]. Concentrates containing both vWF and factor VIII can be administered on the day of the surgery, and levels should be checked preoperatively [13,14]. With Wilfactin and rvWF, however, time needs to be given for factor VIII stabilization; therefore, administration should be 12 to 24 hours preoperatively [13,14]. In both cases, additional treatment should be administered if required. As these concentrates have short half-lives, additional doses during the postoperative period are required [13,14].

If standard vWD management strategies are not sufficient, platelet infusion should be considered. Another rescue measure is recombinant factor VIIa, which has been successfully used in vWD patients who developed alloantibodies to pdvWF [13].

Immune thrombocytopenia: pathophysiology and presentation

ITP is an acquired autoimmune type of thrombocytopenia (platelet count <150,000/mL) that results from peripheral destruction and impaired megakaryopoiesis [7-10]. Platelet destruction is thought to be mediated by the loss of tolerance to platelet glycoproteins, with an immune T-cell-dependent response that leads to the production of IgG autoantibodies against platelet proteins, and their subsequent destruction by phagocytes. Impaired megakaryopoiesis occurs due to the autoimmune targeting megakaryocytes [7-10]. This condition can be divided into primary (idiopathic) or secondary, with the latter being secondary to another disorder (autoimmune disorder, viral infection, immunological dysfunction, as in lymphoma and leukemia) [7,9,10].

As in other forms of thrombocytopenia, the main manifestation is bleeding, ranging from minor bleeding to severe life-threatening hemorrhage, depending on the absolute platelet count and comorbid conditions [7,11]. ITP is a diagnosis of exclusion, requiring that other causes of thrombocytopenia be refuted [7,8,11].

Immune thrombocytopenia: bleeding risk and treatment

The bleeding risk in thrombocytopenic patients often correlates with the absolute platelet count: the risk of epistaxis, petechiae, or ecchymoses increases from counts below 20,000/mL, spontaneous mucosal bleeding often occurs under 10,000/mL, and severe internal bleeding may ensue below 5,000/mL [7,12]. However, in ITP, the said correlation is weaker, with evidence showing that ITP patients tend to bleed less commonly at comparable platelet counts (possibly from a compensatory increase in platelet function and size) [9,11]. Furthermore, the bleeding risk is also affected by a myriad of other factors, such as age, older patients tend to bleed more, comorbid conditions, and the administration of medications, fluids, and blood products [11,12].

As the bleeding risk varies, ITP treatment must be tailored to each patient, namely, to their global hemostatic status, age, comorbidities and medications predisposing to bleeding, need for interventions with increased bleeding risk, activity, and lifestyle [11]. According to current guidelines, treatment is rarely indicated for patients with counts above 20,000 /mL in the absence of bleeding, with exceptions being made for surgical patients and those in need of antiplatelet or anticoagulation therapy [11]. Treatment options include corticosteroids, intravenous immunoglobulins (IVIgs), anti-D immunoglobulin, platelet transfusion, splenectomy, rituximab, and thrombopoietin receptor agonists [9,11]. Corticosteroids are the standard initial treatment for adults with ITP [11]. IVIg may be appropriate in patients with bleeding, at high risk for bleeding, who require a surgical procedure, or who are unresponsive to corticosteroids [11]. In life-threatening bleeding, a treatment combination is recommended, including intravenous corticosteroids, IVIg, and platelet transfusion; notably, plasmapheresis and recombinant factor VIIa are not recommended [11]. Antifibrinolytic agents may be useful in preventing bleeding associated with surgical procedures [9,11].

Anesthetic considerations for von Willebrand disease and immune thrombocytopenia patients

Preoperative Evaluation, Testing, and Optimization

Perioperative management of both vWD and ITP by a multidisciplinary team, including the surgeon, anesthesiologist, hematologist, and transfusion medicine expert, is highly recommended to draft a tailored approach [11-13,20]. In fact, vWD patients managed in hemostasis specialized centers seem to have a better prognosis [13,15]. A detailed history should be taken, including history of prior bleeding and response to implemented measures (desmopressin, recombinant factors, and blood transfusions for vWD and corticosteroids, IVIg, and platelet transfusions for ITP) [12,13,26].

Apart from standard preoperative studies, vWD patients should be assessed for their vWF and factor VIII levels, with FVIII:C, vWF:Ag and vWF:RCo tests [6,13,27]. For major surgery, most sources recommend maintaining vWF:RCo and FVIII:C levels of 100 IU/dL or higher in the preoperative period [6,13,14,20,27]. For minor surgery and invasive procedures, the literature is more heterogeneous, but safe thresholds are established around 30-50 IU/dL for both vWF:RCo and FVIII:C [6,13,14,20,27]. If the patient’s levels do not meet these thresholds, they should be corrected preoperatively [6,13,14,20,27]. Additionally, levels should be reassessed at least two hours before surgery to allow for additional treatment if required [14]. Table 1 summarizes the perioperative management of vWD strategies proposed in the referenced literature.

**Table 2:** Summary of vWD management strategies. ^*^: Only if the patient’s levels require correction; ^a^: IV DDAVP dose: 0.3 µg/kg (maximum 20 µg); ^b^: tailored dose: desired increase in factor level × weight in kg/²; ^c^: TXA dose: 10-15 mg/kg. vWF = von Willebrand factor; RCo = ristocetin cofactor; FVIII = factor VIII; DDAVP = desmopressin; TXA = tranexamic acid

Type of surgery	Timing	Target vWF:RCo/FVIII levels (IU/dL)	vWF and FVIII levels correction*		Adjunctive TXA
			DDAVP responsive	DDAVP non-responsive	
Minor	Preoperative	30–50 IU/dL	IV DDAVP^a^ (90 minutes prior)	vWF concentrate^b^ (~2 hours prior; or 12–24 hours for FVIII-less concentrates)	TXA 1 g
	Postoperative	>30 IU/dL for 1–5 days	IV DDAVP^a^ every 8 or 12 hours	vWF concentrate daily^b^	TXA^c^ 8/8 hours
Major	Preoperative	≥100 IU/dL	IV DDAVP^a^ (90 minutes prior)	vWF concentrate^b^ (~2 hours prior; or 12–24 hours for FVIII-less concentrates)	TXA 1 g
	Postoperative	>50 IU/dL for 7–14 days	IV DDAVP^a^ every 8 or 12 hours (if tachyphylaxis: vWF concentrate^b^)	vWF concentrate daily^b^	TXA^c ^8/8 hours
Acute major bleeding/Emergent surgery		≥100 IU/dL	vWF concentrate (containing FVIII): 40–60 IU/kg		TXA 1 g

For ITP, current consensus-based guidelines recommend treatment aiming for a platelet count of >50,000/mL for minor surgery, >80,000/mL for major surgery, and >100,000/mL for neurosurgery [11]. As previously stated, corticosteroids are the standard initial treatment; however, additional treatments may be indicated or necessary in the context of urgent or emergent surgery [11].

Provided the patient has no contraindication, the administration of 1 g tranexamic acid immediately before the surgery is recommended by most sources, both alone and as an adjunctive therapy for vWD [14,21,27], and can also be beneficial for ITP patients [9,11].

Our patient presented with both vWD and severe thrombocytopenia, presenting a high bleeding risk in an already frail patient. Preoperative management of the case followed the recommendations mentioned above. Notably, a multidisciplinary team, including Orthopedics, Anesthesia, Hematology, and Transfusion Medicine specialists, was assembled, ITP-directed therapy was successfully initiated, and vWF and factor VIII levels were controlled preoperatively. Surgery was delayed until platelet level optimization, with both platelet infusion and corticosteroid therapy being applied. Given that our patient had levels above the recommended, no factor-level correction was necessary, and only tranexamic acid was administered as bleeding prophylaxis. It is also worth noting that tranexamic acid administration was decided, even though our patient had a prior history of thrombotic events, once the hemorrhagic risk was weighed against the thrombotic risk.

Anesthetic Technique and Intraoperative Concerns

In vWD patients, given the risk for hemorrhagic complications, especially with neuraxial techniques, general anesthesia is normally favored over regional anesthesia [13,21]. Even though there are no specific guidelines about the use of regional anesthesia in patients with coagulation disorders, there have been some reports of successful cases in both hemophilia and vWD patients [28-30]. As such, it is considered reasonable to use it as an option for selected patients after careful consideration of the individual risk-benefit ratio (based on vWD subtype, severity, and vWF:RCo and FVIII:C levels at the time of the procedure) and patient consent [6,13]. Most literature, including two vWD management guidelines, recommends vWF:RCo levels higher than 50 IU/dL to safely perform regional anesthesia [6]; nevertheless, some sources contraindicate neuraxial anesthesia in type 2 and 3 vWD [14].

Likewise, thrombocytopenia also carries risk for hemorrhagic complications; therefore, most available clinical guidance on anesthesia in thrombocytopenic patients recommends a platelet count above 75,000-80,000/mL for safe neuraxial anesthesia, as well as a platelet count re-check before the blockade [31-33]. Remarkably, in ITP, some sources allow for the use of neuraxial techniques if the platelet count is >50,000/mL, by experienced anesthesiologists and after a careful risk‐benefit assessment, as the platelet function is normal or even augmented [31,32].

Perioperative avoidance of trauma is paramount in patients with bleeding disorders, including vWD and ITP patients, namely, by careful airway management, preventing traumatic intubation, cautious positioning and mobilization, pressure point care, avoiding intramuscular injections, and cautious placement of vascular lines, preferably under ultrasound guidance [7,13]. With the intention of avoiding further compromise of coagulation and hemostasis, it is fundamental to maintain normothermia and avoid drugs that increase the hemorrhagic risk, such as NSAIDs [13].

As suggested, we opted to avoid neuraxial anesthesia, with general anesthesia being used. NSAIDs were also avoided, with analgesia being complemented with an ultrasound-guided peripheral nerve block, as the risk profile is more favorable for peripheral regional anesthesia. During the procedure, efforts were made to maintain normothermia and avoid trauma as much as possible. Given the hemorrhagic risk and the patient’s preexisting anemia, we foresaw the need for serial blood gas, hemoglobin, and coagulation assays and invasive blood pressure control, so an arterial line was placed with minimal trauma. Lastly, the management strategies implemented were successful, as blood loss was relatively limited.

Postoperative Period Care and Monitoring

The hemostatic challenge initiated during surgery carries through the postoperative period; hence, it is recommended to maintain ITP-directed treatment and vigilance over the platelet count, as well as to monitor and vWF:RCo and FVIII:C values at least daily. Most sources recommend keeping levels above 50 IU/dL for major surgery and 30 IU/dL for minor surgery, for 7-14 days and 1-5 days, respectively [6,13,14,27].

There have been reports of venous thromboembolism in patients given FVIII and/or vWF concentrates, with some studies proposing elevated plasma FVIII:C levels at least contributing to its etiology [13,27]. Thus, it is proposed that patients who are considered at risk of thrombosis (by their co-existing diseases, type of surgery, or high FVIII:C levels above 150-250 IU/dL) be given low-dose heparin prophylaxis postoperatively and continued until the end of factor replacement [13,14,21,27]. Besides, early mobilization and compression stockings should also be employed [21].

The postoperative period was the moment in which the case further deviated from the recommended management principles: there was no control of factors’ levels after the surgery, tranexamic acid was only administered for 24 hours postoperatively, and in a reduced dose (because of the coexisting thrombotic risk). In fact, despite no clinically apparent hemorrhage, there was a significant postoperative hemoglobin drop, requiring the transfusion of a total of three units of RBC. A tighter postoperative control might have partially prevented the occult blood loss and transfusion need. Nonetheless, globally, the case had a positive outcome, as no life-threatening hemorrhage occurred and the patient was safely discharged seven days after surgery.

## Conclusions

vWD and ITP are primary hemostasis disorders that carry an important hemorrhagic risk, requiring a complex multidisciplinary management. It is essential to understand not only their pathophysiology and therapeutic agents, but also the patient’s individual bleeding phenotype and prior response to said agents, so that a tailored care plan can be instituted. Trauma minimization, vWF and factor VIII levels, and platelet count monitoring and correction throughout the entire perioperative period are vital for a positive outcome. Lastly, it is important to remember that the postoperative period is as important as the earlier phases, as the hemostatic challenge has not yet resolved, and significant bleeding can occur, as our case illustrates.
